# Hypoxia signaling pathways: modulators of oxygen-related organelles

**DOI:** 10.3389/fcell.2015.00042

**Published:** 2015-07-21

**Authors:** Miriam J. Schönenberger, Werner J. Kovacs

**Affiliations:** Department of Biology, Institute of Molecular Health SciencesETH Zurich, Zurich, Switzerland

**Keywords:** endoplasmic reticulum, ER stress, hypoxia, HIF-α, mitochondria, mitophagy, peroxisomes, pexophagy

## Abstract

Oxygen (O_2_) is an essential substrate in cellular metabolism, bioenergetics, and signaling and as such linked to the survival and normal function of all metazoans. Low O_2_ tension (hypoxia) is a fundamental feature of physiological processes as well as pathophysiological conditions such as cancer and ischemic diseases. Central to the molecular mechanisms underlying O_2_ homeostasis are the hypoxia-inducible factors-1 and -2 alpha (HIF-1α and EPAS1/HIF-2α) that function as master regulators of the adaptive response to hypoxia. HIF-induced genes promote characteristic tumor behaviors, including angiogenesis and metabolic reprogramming. The aim of this review is to critically explore current knowledge of how HIF-α signaling regulates the abundance and function of major O_2_-consuming organelles. Abundant evidence suggests key roles for HIF-1α in the regulation of mitochondrial homeostasis. An essential adaptation to sustained hypoxia is repression of mitochondrial respiration and induction of glycolysis. HIF-1α activates several genes that trigger mitophagy and represses regulators of mitochondrial biogenesis. Several lines of evidence point to a strong relationship between hypoxia, the accumulation of misfolded proteins in the endoplasmic reticulum, and activation of the unfolded protein response. Surprisingly, although peroxisomes depend highly on molecular O_2_ for their function, there has been no evidence linking HIF signaling to peroxisomes. We discuss our recent findings that establish HIF-2α as a negative regulator of peroxisome abundance and suggest a mechanism by which cells attune peroxisomal function with O_2_ availability. HIF-2α activation augments peroxisome turnover by pexophagy and thereby changes lipid composition reminiscent of peroxisomal disorders. We discuss potential mechanisms by which HIF-2α might trigger pexophagy and place special emphasis on the potential pathological implications of HIF-2α-mediated pexophagy for human health.

## Introduction

Life with oxygen (O_2_) began around 2.4 billion years ago, when photosynthetic organisms prospered and multiplied, leading to a progressive increase of atmospheric O_2_. O_2_-related organelles, such as mitochondria, peroxisomes, and plastids, must have been acquired after that date (De Duve, 2007; Semenza, 2007). The appearance of O_2_ was one of the defining moments in evolution, as it offered organisms the advantage of generating energy more efficiently. Because of the high energy potential of O_2_, aerobic organisms have become dependent on this gas for their performance and survival. O_2_ is an essential substrate in cellular metabolism, bioenergetics, and signaling and as such inseparably linked to the survival and normal function of all metazoans. Hence, aerobic species developed mechanisms to sense O_2_ levels and regulate O_2_ consumption, in order to cope with conditions of insufficient O_2_ supply. This Review focuses on the role of hypoxia-inducible factors (HIFs) as master regulators of O_2_ homeostasis and, in particular, on recent advances in understanding their roles in regulating major O_2_-consuming organelles, namely mitochondria, the endoplasmic reticulum (ER), and peroxisomes.

## Regulation of HIFs

**Figure 1:**
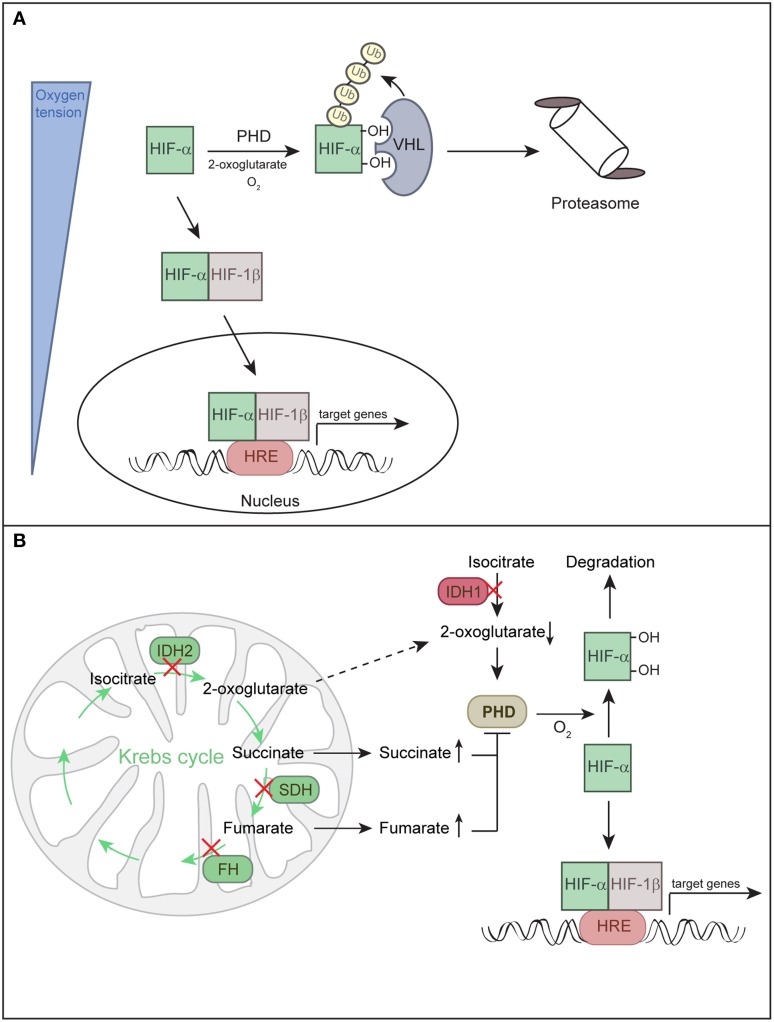
**Regulation of HIF-α subunits. (A)** Hypoxia-inducible factors (HIFs) are transcription factors composed of O_2_-regulated α subunits (HIF-1α or HIF-2α) and a constitutively expressed HIF-1β subunit. Together these subunits bind hypoxia response elements (HRE) to mediate adaptive responses to hypoxia. HIF-α activity is directly linked to oxygen partial pressure. Under normoxia, HIF-α is hydroxylated by prolyl hydroxylase domain protein (PHD) and targeted for proteasomal degradation by the von Hippel-Lindau (VHL) E3 ubiquitin ligase complex. Under hypoxia, hydroxylation is inhibited and HIF-α is stabilized, it dimerizes with HIF-1β and enters the nucleus to induce target gene transcription. **(B)** HIF-α can be stabilized irrespective of O_2_ tension due to inhibition of PHDs, a state defined as pseudohypoxia. Mutations in the Krebs cycle enzymes succinate dehydrogenase (SDH) and fumarate hydratase (FH) lead to accumulation of succinate and fumarate, respectively, whereas mutations in isocitrate dehydrogenases 1/2 (IDH1 and IDH2) lead to low levels of 2-oxoglutarate. Succinate and fumarate inhibit PHDs, while low levels of the co-substrate 2-oxoglutarate decrease the activity of PHDs. Decreased activity of PHDs leads to a low rate of HIF-α hydroxylation under normoxic conditions and stabilization of HIF-α.

Central to the molecular mechanisms underlying O_2_ homeostasis are HIF-1α and HIF-2α that function as master regulators of the adaptive response to hypoxia. HIFs form a heterodimer consisting of a constitutively expressed ARNT/HIF-1β subunit and O_2_-regulated α subunits (HIF-1α or EPAS1/HIF-2α) (Majmundar et al., 2010; Keith et al., 2012). A third HIF-α subunit (HIF-3α) has also been described. *HIF3A* mRNA is differentially spliced to produce multiple HIF-3α isoforms that either promote or inhibit the activity of other HIF complexes (Keith et al., 2012). Under normoxia, HIF-α subunits are hydroxylated by prolyl hydroxylases (PHD1-3) and recognized and targeted for proteasomal degradation by the von Hippel-Lindau (VHL) E3 ubiquitin ligase complex (Figure 1A). PHD enzymes are 2-oxoglutarate- and iron-dependent dioxygenases, whose activity is absolutely dependent on O_2_. Hence, the rate of HIF-α hydroxylation is suppressed under hypoxia. Hypoxia or loss of functional VHL stabilizes HIF-α subunits. HIF-α either dimerizes with HIF-1β and binds to hypoxia-responsive elements in promoters of target genes to promote a concerted transcriptional response (Keith et al., 2012) (Figure 1A) or it physically interacts with other non-HIF proteins (Uniacke et al., 2012; Hubbi et al., 2013), enabling convergence of HIF O_2_ sensing with other signaling pathways.

HIF-α subunits can also be stabilized under non-hypoxic conditions, a phenomenon termed “pseudohypoxia.” In addition to O_2_, PHDs are sensitive to changes in certain Krebs cycle intermediates. Mutations in four genes involved in the metabolism of citrate have the potential to stabilize HIF-α by inhibiting HIF-α hydroxylation and are linked to various tumors (Figure 1B) (Raimundo et al., 2011; Losman and Kaelin, 2013). Succinate dehydrogenase and fumarate hydratase deficiencies lead to accumulation of succinate and fumarate, respectively, and these metabolites compete with 2-oxoglutarate to inhibit PHDs. Mutations in isocitrate dehydrogenases 1 and 2 could promote HIF-α stabilization as a result of low levels of 2-oxoglutarate, which is an essential co-substrate of PHDs (Thompson, 2009). Tumor-associated isocitrate dehydrogenase mutations cause a gain of function, leading to high-level production of (*R*)-2-hydroxyglutarate and depletion of 2-oxoglutarate (Losman and Kaelin, 2013). PHD enzymes were initially reported to be inhibited by (*R*)-2-hydroxyglutarate (Zhao et al., 2009). However, further studies have shown that (*R*)-2-hydroxyglutarate potentiates PHD activity and blunts the induction of HIF-α in response to hypoxia (Losman and Kaelin, 2013).

HIF-1α is expressed ubiquitously, whereas HIF-2α is selectively expressed in distinct cell populations of most organs (Majmundar et al., 2010). HIF-1α and HIF-2α have both overlapping and distinct target genes (Keith et al., 2012) and they are differentially regulated in various physiological settings (e.g., embryonic development) and function in pathophysiological conditions such as cancer and ischemic diseases (Semenza, 2012). They have also different roles in tumorigenesis dependent on specific tumor microenvironments (Majmundar et al., 2010; Keith et al., 2012). HIF-induced transcription promotes angiogenesis, erythropoiesis, metastasis and metabolic reprogramming, such as shifting cell metabolism from oxidative phosphorylation to glycolysis. HIF activation due to hypoxia or loss of VHL function also reprograms lipid metabolism leading to lipid accumulation (Huss et al., 2001; Boström et al., 2006; Rankin et al., 2009; Kucejova et al., 2011; Qu et al., 2011; Walter et al., 2014).

## Mitochondria and HIF-α

Mitochondria, metabolism, and O_2_ are inextricably intertwined. In the following sections we will discuss the numerous mechanisms by which HIF signaling can affect mitochondrial function.

### HIF-dependent regulation of mitochondrial metabolism

**Figure 2:**
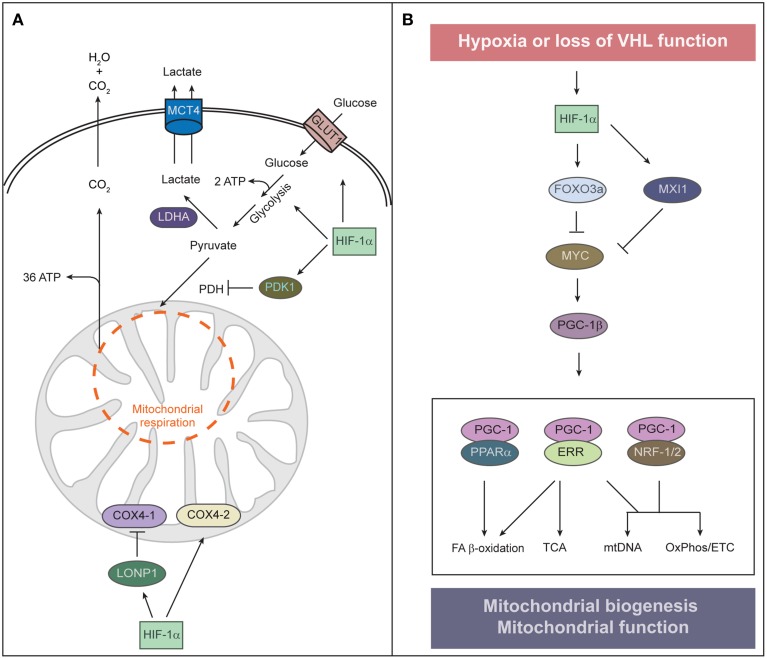
**Regulation of mitochondrial function and abundance by HIF-α. (A)** To adapt to low oxygen tension, cells undergo two HIF-1α-mediated alterations of cellular metabolism: O_2_-independent ATP production and reduction of mitochondrial O_2_ consumption. HIF-1α signaling also contributes to the Warburg effect of aerobic glycolysis—that is, an uncoupling of glycolysis from O_2_ levels—by stimulating the expression of the glucose transporter GLUT1 and glycolytic enzymes. Increased glycolysis generates increased levels of pyruvate, which is largely converted to lactate by HIF-inducible lactate dehydrogenase A (LDHA) and removed from the cell by the monocarboxylate transporter 4 (MCT4). HIF-1α induces pyruvate dehydrogenase kinase 1 (PDK1), which inhibits pyruvate dehydrogenase (PDH) and blocks conversion of pyruvate to acetyl-CoA, resulting in decreased flux through the tricarboxylic acid (TCA) cycle. Decreased TCA cycle activity attenuates oxidative phosphorylation and excessive mitochondrial ROS production. Under normoxia, COX4-1 is the predominant isoform of COX4 present in complex IV of the electron transport chain, which transfers electrons to O_2_. Under hypoxia, HIF-1α upregulates the expression of *COX4-2* and the mitochondrial protease *LONP1*, which in turn degrades COX4-1. COX4-2 is more efficient at facilitating the electron transfer to O_2_ and thereby protects the cell from oxidative damage during hypoxia. **(B)** Control of mitochondrial biogenesis by HIF-α. HIF-1α induces the expression of MAX-interacting protein 1 (MXI1), a repressor of MYC activity, and thereby represses a subset of MYC target genes such as *PGC-1*β. HIF-1α-dependent activation of FOXO3a inhibits MYC activity by reducing MYC protein stability. Interaction between PGC-1 and transcription factors such as PPARα, ERR, and NRF-1/2 orchestrates the major functions of mitochondria. HIF-1α-mediated inhibition of MYC and PGC-1 results in reduced mitochondrial biogenesis.

The hypoxia-dependent increase in the abundance and activity of HIF-1α and the HIF-1α-dependent transcriptional program have three major effects on metabolism that serve to equilibrate O_2_ consumption with O_2_ supply. First, HIF-1α promotes glycolytic energy production by inducing genes that encode glucose transporters (e.g., *GLUT1, GLUT3*) and glycolytic enzymes (Figure 2A) (Semenza, 2010). HIF-1α also upregulates lactate dehydrogenase A (LDHA), which converts pyruvate to lactate and regenerates NAD^+^ for continuous supply for glycolysis, and monocarboxylate transporter 4 (MCT4), which transports lactate out of the cell (Figure 2A).

Second, HIF-1α suppresses both the Krebs cycle and oxidative phosphorylation within mitochondria. HIF-1α induces pyruvate dehydrogenase kinase 1 (PDK1), which phosphorylates and inactivates the mitochondrial enzyme pyruvate dehydrogenase that catalyzes the conversion of pyruvate to acetyl-CoA, thereby shunting pyruvate away from mitochondria and diminishing hypoxic mitochondrial respiration (Kim et al., 2006; Papandreou et al., 2006). A benefit of attenuating mitochondrial respiration under hypoxia is reducing the amount of toxic mitochondrial reactive oxygen species (mtROS) generated by inefficient respiration. Mouse embryonic fibroblasts lacking *Hif1*α undergo cell death as a result of excess mtROS production due to a failure of PDK1 induction (Kim et al., 2006). HIF-1α also reduces mtROS production under hypoxic conditions by optimizing respiration efficiency through inducing cytochrome c oxidase (COX) subunit IV isoform 2 (COX4-2) and the mitochondrial protease LONP1, which degrades the less efficient COX4-1 (Fukuda et al., 2007). Mitochondrial ROS also modulate cell signaling through stabilization of HIF-1α, due to PHD inhibition (Sena and Chandel, 2012). Cells utilize an acute increase in mtROS to stabilize HIF under hypoxia and subsequently restrain ROS production under chronic hypoxia to avoid cellular damage. ROS have a relatively short diffusion distance and thus, mtROS signaling may rely on proximity of ROS-producing mitochondria to their sites of action. Intriguingly, perinuclear clustering of mitochondria triggered by hypoxia is accompanied by the accumulation of nuclear ROS and required for maximal HIF-1α binding to the *VEGF* promoter and *VEGF* expression (Al-Mehdi et al., 2012).

Third, hypoxia shifts mitochondrial glutamine metabolism from oxidation to reductive carboxylation (Sun and Denko, 2014). The activity of the α-ketoglutarate dehydrogenase complex is decreased under hypoxia, since HIF-1α induces the E3 ubiquitin ligase SIAH2 that mediates the proteasomal degradation of the E1 subunit of α-ketoglutarate dehydrogenase. Increased α-ketoglutarate levels drive the reverse reaction at isocitrate dehydrogenase, and the glutamine-derived citrate can be transported to the cytoplasm in order to generate acetyl-CoA for anabolic processes under hypoxia.

Cancer cells exhibit a high rate of glycolysis even in the presence of O_2_, a phenomenon known as aerobic glycolysis or the “Warburg effect” (Vander Heiden et al., 2009). HIF-1α activation as a result of *VHL* loss or pseudohypoxia contributes also to the development of the Warburg effect. Furthermore, although the shift from oxidative phosphorylation to glycolysis is attributed to the activity of HIF-1α, at least in the liver HIF-2α induces the same genes involved in this metabolic adaptation (Rankin et al., 2009; Walter et al., 2014).

### Mitochondrial biogenesis and HIF-α

A second key remodeling of mitochondria in hypoxia is suppression of mitochondrial biogenesis to decrease mitochondrial mass and O_2_ consumption. Mitochondrial biogenesis involves replication of the mitochondrial genome and coordinated expression of nuclear- and mitochondrial-encoded gene products. It depends upon the activity of a hierarchy of nuclear transcription factors that includes the peroxisome proliferator-activated receptor (PPAR) γ coactivator 1 family of transcriptional coactivators [PGC-1α, PGC-1β, and the PGC-related coactivator (PRC)], the nuclear respiratory factors (NRF1 and NRF2), and estrogen-related receptors (ERRα, ERRβ, and ERRγ) (Figure 2B) (Scarpulla et al., 2012). PGC-1α is the master regulator of all aspects of mitochondrial biogenesis, including activation of respiratory chain and fatty acid oxidation genes, increased mitochondrial number, mtDNA replication, and augmentation of mitochondrial respiratory capacity. It exerts these effects through direct interaction with and coactivation of PPARs, NRFs, and ERRs (Scarpulla et al., 2012). Nutrient supply and cellular energy balance regulate the activity of PGC-1α at both the transcriptional and posttranslational level (Dominy et al., 2010; Scarpulla et al., 2012). PGC-1β and PRC interact with and coactivate many of the same transcription factors as PGC-1α to promote mitochondrial biogenesis (Scarpulla et al., 2012).

The oncogenic transcription factor MYC promotes mitochondrial biogenesis through activation of PGC-1β (Li et al., 2005; Zhang et al., 2007). MYC dimerizes with MYC-associated factor X (MAX) and binds specific E-box sequences in target gene promoters to activate transcription. Heterodimers of MAX with MAX-interacting protein 1 (MXI1) antagonize MYC function and repress transcription by binding to the same promoter regions of MYC target genes. Cross-talk between HIF and MYC has been defined at a number of levels, however, HIF-1α and HIF-2α exert opposing roles on MYC interaction with its transcription cofactors (Dang et al., 2008; Keith et al., 2012). HIF-1α activation inhibits mitochondrial biogenesis by promoting MYC degradation and by inducing MXI1 expression (Zhang et al., 2007). This inhibition is mediated by the transcription factor FOXO3a (Forkhead-box protein O3a) which is activated in hypoxia downstream of HIF-1α (Peck et al., 2013). FOXO3a can also inhibit MYC activity by reducing MYC protein stability and by increasing the expression of miRNAs that perturb the translation of *MYC* mRNA (Peck et al., 2013). Huang et al. (2014) reported that HIF-1α, but not HIF-2α, represses MYC in human hepatoma cells and thereby decreases *PGC-1*β expression, leading to decreased expression of medium- and long-chain acyl-CoA dehydrogenases and subsequent inhibition of mitochondrial fatty acid β-oxidation. It has not been addressed if HIF-1α-mediated downregulation of PGC-1β also affects mitochondrial biogenesis and mass in hepatoma cells. HIF-2α actually enhances MYC transcriptional activity by binding to and stabilizing the MAX-MYC heterodimer (Gordan et al., 2007, 2008). The cooperation of HIF-2α with MYC increases MYC effects on various cell cycle regulators and drives tumorigenesis. Given the opposite effects of HIF-1α and HIF-2α on MYC, it remains to be established how MYC is modulated in cells that express both HIF isoforms.

However, with the exception of HIF-1α-mediated inhibition of PGC-1β, data about HIF-α mediated regulation of PGC-1α, the master regulator of mitochondrial biogenesis and function, are relatively scarce. Several studies demonstrated an induction of PGC-1α by hypoxia independently of HIF-1α activity (Shoag and Arany, 2010). Overexpression of PGC-1α under normoxia induces mitochondrial biogenesis which increases O_2_ consumption and decreases intracellular O_2_ levels, leading to HIF-1α protein stabilization and activation of HIF-1α target genes (O'Hagan et al., 2009). ROS accumulation in hypoxic cancer cells induces expression of *PGC-1*α*/*β to promote detoxification through induction of antioxidative enzymes (Austin and St-Pierre, 2012). The precise relationship between the PGC-1α and HIF-α pathways with respect to mitochondrial biogenesis obviously needs further clarification.

### HIF-dependent regulation of mitophagy

The third mechanism by which HIF-α controls mitochondrial function is selective mitochondrial autophagy (mitophagy). Autophagy is an evolutionary conserved catabolic process for degradation of macromolecules and organelles. Both non-selective “bulk” autophagy and selective autophagy of specific proteins or organelles have been described (Mizushima et al., 2011; Schreiber and Peter, 2014). Selective and non-selective autophagy share a set of autophagy-related (Atg) proteins referred to as the core autophagic machinery (Stolz et al., 2014). Yeast Atg8 and its mammalian homologs of the microtubule-associated protein-1 light chain 3 family (LC3A, LC3B, LC3C) and γ-aminobutyric acid-receptor-associated (GABARAP, GABARAPL1, GABARAPL2) proteins are covalently conjugated to the lipid phosphatidylethanolamine upon induction of autophagy. Besides playing a pivotal role in different steps of autophagosome biogenesis, the LC3 family members are important for target recognition during selective autophagy. Selective autophagy requires specific receptors, which recognize cargo tagged with degradation signals, connect it to the autophagosomal membrane through their LC3-interacting regions (LIR), and are degraded together with their cargo within autolysosomes (Stolz et al., 2014).

Mitophagy can be initiated in several ways to arbitrate mitochondrial quality control via the selective removal of superfluous or damaged mitochondria. The best-studied mechanism for mitophagy in mammalian cells is the PINK1-Parkin-mediated pathway, which is elegantly reviewed elsewhere (Scarffe et al., 2014). Briefly, phosphatase and tensin homolog (PTEN)-induced putative protein kinase (PINK1), which is rapidly degraded in healthy mitochondria, accumulates upon mitochondrial membrane depolarization at the outer membrane, leading to the recruitment of the E3 ubiquitin ligase Parkin to mitochondria and ubiquitination of several outer mitochondrial membrane (OMM) proteins (Winklhofer, 2014). The recruitment of ubiquitinated mitochondria to autophagic structures is mediated by LC3 family members and ubiquitin-binding adaptor proteins such as sequestosome 1 (SQSTM1/p62), NBR1 (neighbor of BRCA1 gene 1), and optineurin (Rogov et al., 2014).

**Figure 3:**
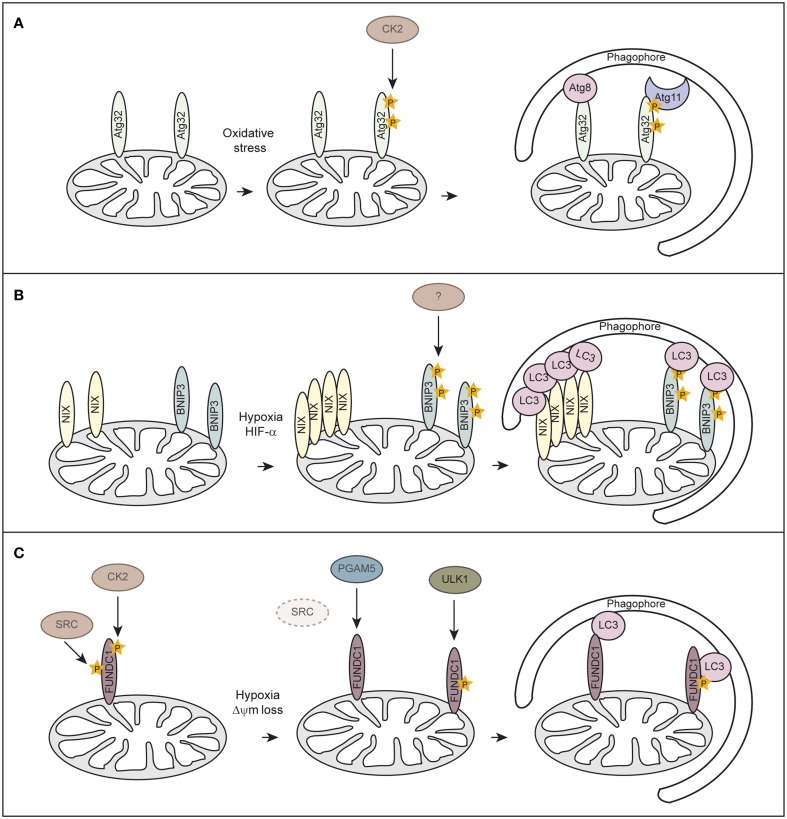
**Receptor-mediated mitophagy. (A)** Atg32-mediated mitophagy in *S. cerevisiae*. Atg32 is an outer mitochondrial membrane protein whose expression is induced upon mitophagy-inducing conditions. Atg32 interacts with Atg8 and Atg11 via distinct domains. Casein kinase 2 (CK2) phosphorylates Atg32 upon mitophagy-inducing conditions, which is essential for the Atg11 interaction without affecting Atg32-Atg8 binding. **(B)** NIX/BNIP3-mediated mitophagy in mammalian cells. NIX and BNIP3 are outer mitochondrial membrane proteins that interact with LC3 through LIR motifs in their N-terminal region. Upon hypoxia, NIX and BNIP3 are transcriptionally induced in a HIF-α-dependent manner. Phosphorylation of BNIP3 promotes its binding to LC3 and subsequent mitophagy. The kinase for BNIP3 phosphorylation is unknown. **(C)** FUNDC1-mediated mitophagy in mammalian cells. FUNDC1 is an outer mitochondrial membrane protein that interacts with LC3 through a LIR domain at its cytosol-exposed N-terminus. Under normal physiological conditions, FUNDC1 is phosphorylated by SRC and CK2, thereby preventing LC3 binding. Upon hypoxia or loss of mitochondrial membrane potential (Δψm), the expression of SRC is strongly suppressed and PGAM5 dephosphorylates FUNDC1. Dephosphorylation of FUNDC1 enhances the interaction between FUNDC1 and LC3 and promotes mitophagy. Phosphorylation of FUNDC1 by ULK1 enhances its binding to LC3.

Although mitophagy has been extensively studied in mammals, mitophagy-specific factors still remain controversial. In yeast Atg32 has been identified as receptor protein for mitophagy (Kanki et al., 2009, 2015; Okamoto et al., 2009). Atg32 localizes to the OMM, harbors a classical LIR consensus sequence and interacts with Atg8 and the scaffold protein Atg11 to enable the assembly of the core autophagy machinery around the cargo (Figure 3A). Casein kinase 2 (CK2) regulates mitophagy by phosphorylating Atg32, which stabilizes the Atg32-Atg11 interaction and promotes mitophagy. So far no mammalian homolog has been identified for Atg32.

Similar to Atg32 in yeast, the mammalian mitophagy receptors BNIP3 (Bcl-2 and adenovirus E1B 19-kDa-interacting protein 3), BNIP3-like (BNIP3L/NIX), and FUNDC1 (FUN14 domain containing 1) are OMM proteins which can directly bind to LC3 via their LIR motifs (Figures 3B,C). BNIP3 and NIX were originally thought to promote apoptosis or programmed necrosis (Zhang and Ney, 2009). They can activate autophagy by binding to Bcl-2 and thereby disrupting the interaction between Beclin-1 and Blc-2/Bcl-X_L_ (Bellot et al., 2009; Boland et al., 2013). BNIP3 and NIX are hypoxia-inducible HIF-α target genes and it has been suggested that they act either in hypoxia-induced macroautophagy or mitophagy (Sowter et al., 2001; Tracy et al., 2007; Zhang et al., 2008; Bellot et al., 2009). While BH3 domains of BNIP3 and NIX are sufficient to induce the general autophagy response (Bellot et al., 2009), induction of mitophagy requires the LIR domain of NIX (Novak et al., 2010) and BNIP3 (Hanna et al., 2012; Zhu et al., 2013). Phosphorylation of BNIP3 at serines flanking its LIR domain promotes binding to LC3 family members and thereby increases mitophagy (Zhu et al., 2013), however, involved kinases are unknown (Figure 3B). It is not clear how phosphorylation of BNIP3 is regulated under hypoxia and if phosphorylation regulates NIX.

Recently, FUNDC1 has been implicated in mediating hypoxia-induced mitophagy (Liu et al., 2012). FUNDC1-mediated mitophagy is regulated at the posttranslational level by reversible phosphorylation. Under normal physiological conditions FUNDC1 is phosphorylated by Src kinase at Tyr18, which is located in the LIR motif, and at Ser13 by CK2 (Figure 3C) (Chen et al., 2014a). In response to hypoxia or loss of mitochondrial membrane potential the mitochondrially localized phosphoglycerate mutase family member 5 (PGAM5), a Ser/Thr phosphatase, dephosphorylates FUNDC1 at Ser13, whereas Tyr18 phosphorylation seems to be prevented before mitophagy-induction due to inactivation of Src kinase (Figure 3C) (Chen et al., 2014a). Dephosphorylated FUNDC1 displays a higher binding affinity to LC3, resulting in selective autophagosome incorporation and autophagic degradation of mitochondria (Liu et al., 2012; Chen et al., 2014a). Moreover, a study showed that hypoxia or mitochondrial uncouplers elevate protein levels of the autophagy-initiating kinase ULK1 (UNC51-like kinase 1) and target ULK1 to damaged mitochondria where it phosphorylates FUNDC1 at Ser17 and thereby enhances FUNDC1 binding to LC3 (Figure 3C) (Wu et al., 2014). However, it remains to be determined how BNIP3, NIX and FUNDC1 are interconnected or even needed during hypoxia-induced mitophagy.

### Hepatic HIF-α signaling and mitochondrial abundance

We examined the effect of HIF-α signaling on hepatic mitochondrial abundance in control and liver-specific *Vhl*^−∕−^, *Vhl*^−∕−^*/Hif1*α^−∕−^, and *Vhl*^−∕−^*/Hif2*α^−∕−^ mice. Mitochondrial protein levels are similar in control and knockout livers (Walter et al., 2014), suggesting that constitutive HIF-α signaling does not affect hepatic mitochondrial mass. However, subcellular fractionation of livers from control and *Vhl*^−∕−^ mice shows that mitochondrial protein levels are decreased in the heavy mitochondrial fraction of *Vhl*^−∕−^ livers compared with controls, whereas their levels are increased in the light mitochondrial fraction (Walter et al., 2014). Further purification of the light mitochondrial fraction by density gradient centrifugation and immunoblots of gradient fractions show that the OMM protein VDAC shifts toward lower density fractions. In summary, HIF-α signaling in the liver alters the ratio between heavy and light mitochondria, whereas mitochondrial protein levels are not changed in whole liver homogenates. It remains to be clarified if and how hepatic HIF-α signaling affects mitochondrial size, ultrastructure and function. Mitochondrial morphology and ultrastructure depend on mitochondria-shaping proteins that regulate organellar fusion and fission (Mishra and Chan, 2014). Rates of mitochondrial fission and fusion respond to changes in metabolism, and mitochondria regulate their shape to adjust their activity with metabolic conditions (Mannella, 2006).

## Endoplasmic reticulum and hypoxia

### ER stress and hypoxia

The ER is an extensive intracellular membrane network that extends throughout the cytoplasm and is essential for the translation and folding of membrane and secretory proteins (Gidalevitz et al., 2013). It is also a critical site of lipid and glucose metabolism, calcium homeostasis, and detoxification of drugs and metabolic byproducts. A biochemical process that is crucial for ER protein homeostasis is the formation of disulfide bridges, which is referred to as oxidative protein folding (Eletto et al., 2014). Disulfide bond generation by ER-localized protein disulfide isomerases is an oxidative process, and molecular O_2_ and H_2_O_2_ are the principal electron acceptors for oxidative folding in the ER (Eletto et al., 2014). Protein disulfide isomerases catalyze oxidation by coupling *de novo* disulfide formation to the reduction of O_2_ to H_2_O_2_.

The term “endoplasmic reticulum stress” defines any perturbation that compromises the protein folding functionality of the ER (Walter and Ron, 2011). A number of biochemical and physiologic stimuli, such as perturbation in calcium homeostasis or redox status, elevated secretory protein synthesis, expression of misfolded proteins, glucose deprivation, altered glycosylation, viral infection, and excess lipids can disrupt ER homeostasis and impose stress to the ER. These disturbances trigger an adaptive signaling pathway known as the unfolded protein response (UPR) that aims to restore ER homeostasis and function. Hypoxia is a physiologically important ER stress common to solid tumors. Several lines of evidence point to a strong relationship between hypoxia and the accumulation of misfolded proteins in the ER (Koumenis et al., 2007). In tumors, hypoxia is also associated with other conditions that can cause ER stress, such as glucose and amino acid deprivation, and oxidative stress.

### The unfolded protein response signaling pathways

**Figure 4:**
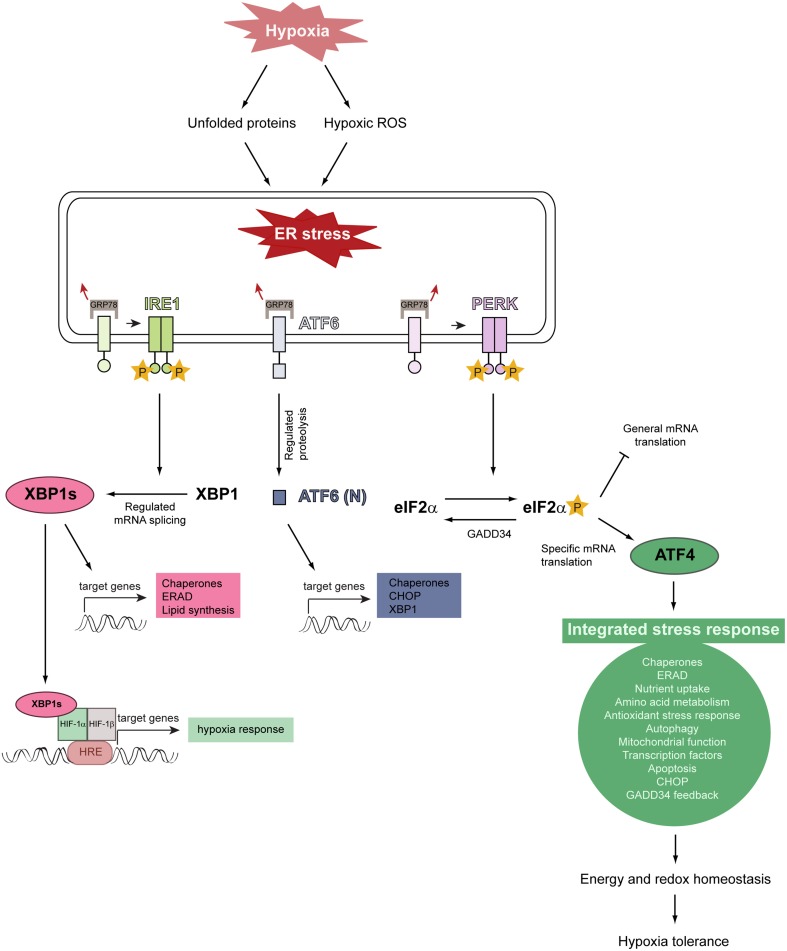
**Model illustrating the relationship between hypoxia, ER stress, and activation of the unfolded protein response**. Severe hypoxic stress perturbs and reduces O_2_-dependent protein folding capacity, resulting in the accumulation and aggregation of misfolded proteins in the ER lumen. Hypoxia increases intracellular ROS production and ROS stimulate multiple biological responses during O_2_ deprivation. Unfolded proteins and hypoxic ROS trigger ER stress which leads to HIF-α-independent activation of the UPR. The UPR is initiated by the stress-sensing ER transmembrane proteins PERK, IRE1, and ATF6. The chaperone GRP78 is normally bound to these ER stress sensors and keeps them inactive, but dissociates from them under ER stress conditions. This dissociation leads to the activation of the three UPR pathways. GRP78 dissociation allows PERK to homodimerize, which facilitates autotransphosphorylation and kinase domain activation. Activated PERK phosphorylates eIF2α which decreases general translation while increasing the preferential translation of specific proteins, such as the transcription factor ATF4. ATF4 triggers the activation of a gene expression program referred to as the integrated stress response. ATF4 induces the expression of *GADD34*, which acts as a negative regulator of the PERK pathway by dephosphorylating eIF2α. The integrated stress response promotes energy and redox homeostasis and is an important prosurvival mechanism under moderate hypoxia. IRE1 homodimerization, followed by autotransphosphorylation, triggers its RNase activity. IRE1-mediated splicing of full-length *XBP1* mRNA generates *XBP1s*, which encodes an active transcription factor. ER stress leads to the translocation of ATF6 to the Golgi, where it is cleaved by regulated intramembrane proteolysis to produce the active transcription factor. Modified from Faust and Kovacs (2014).

Activation of the three canonical branches of the UPR is mediated by three stress-sensing ER transmembrane proteins: protein kinase RNA-like ER kinase (PERK), inositol-requiring protein 1α (IRE1α), and activating transcription factor 6 (ATF6) (Figure 4) (Walter and Ron, 2011; Faust and Kovacs, 2014). In a stress-free ER, these sensors are bound by the ER-resident chaperone glucose-regulated protein of 78 kDa (GRP78) in their intraluminal domains and rendered inactive. Upon ER stress, GRP78 dissociates from PERK, IRE1, and ATF6, leading to their activation (Bertolotti et al., 2000; Shen et al., 2002).

IRE1 is a serine-threonine kinase and endoribonuclease, which catalyzes the splicing of full-length *XBP1* (X-box-binding protein 1) mRNA to generate an active transcription factor, termed spliced *XBP1s*. XBP1s activates genes encoding proteins involved in protein folding, ER-associated degradation (ERAD) of misfolded ER proteins, and lipid synthesis.

ATF6 is comprised of two isoforms, ATF6α and ATF6β, and resides as transcriptionally inactive precursor protein in the ER membrane. ER stress leads to ATF6 translocation from the ER to the Golgi apparatus where it is cleaved sequentially by Site-1 and Site-2 proteases to produce an active transcription factor. ATF6 induces *XBP1* and genes mainly encoding ER chaperones and proteins involved in ERAD.

Dissociation of GRP78 from PERK leads to its homodimerization and activating autophosphorylation. PERK phosphorylates the α-subunit of the eukaryotic translation initiation factor 2 (eIF2α) on serine 51. This phosphorylation event attenuates general translation, resulting in a reduced protein folding load of the ER (Baird and Wek, 2012), but it stimulates selective translation of the activating transcription factor 4 (ATF4), which plays a crucial role for the adaptation to stress. ATF4 target genes are involved in protein folding and assembly, metabolism, nutrient uptake, gene expression, alleviation of oxidative stress, autophagy, and the regulation of apoptosis. In addition to PERK, three other kinases induce eIF2α phosphorylation and preferential translation of ATF4: GCN2 (general control non-derepressible kinase 2), PKR (double-stranded RNA-activated protein kinase), and HRI (heme-regulated inhibitor kinase) (Baird and Wek, 2012). The PERK-eIF2α-ATF4 pathway is referred to as the integrated stress response (ISR), because divergent signals activate the four eIF2α kinases and the ISR, which seeks to remediate stress and restore cellular homeostasis.

### Hypoxia and the unfolded protein response

As protein synthesis and O_2_-dependent protein folding are energy-intensive processes and chronic hypoxia markedly reduces intracellular ATP levels (Kim et al., 2006; Liu et al., 2006), control of mRNA translation is an important cellular response to hypoxia. Hypoxia activates PERK and thereby leads to eIF2α phosphorylation and global translation inhibition, whereas translation of ATF4 is increased in a PERK/eIF2α-dependent manner (Koumenis et al., 2002, 2007; Blais et al., 2004; Bi et al., 2005; Koritzinsky et al., 2006; Wouters and Koritzinsky, 2008). This is a rapid HIF-1α-independent response, occurring within minutes when cells are exposed to anoxic conditions and somewhat more slowly during moderate hypoxia. eIF2α phosphorylation is transient due to the negative feedback loop initiated by ATF4-dependent upregulation of GADD34 (growth arrest DNA-inducible gene 34), which dephosphorylates eIF2α (Figure 4).

Hypoxia increases intracellular ROS production in various cells to stimulate multiple biological responses, and mitochondria appear to be the primary source of hypoxic ROS (Liu et al., 2008). Mitochondrial hypoxic ROS activate the ISR to promote energy and redox homeostasis and to constitute an early adaptive response to hypoxia. Enzymatic antioxidants such as catalase and glutathione peroxidase reduce eIF2α phosphorylation caused by hypoxia, suggesting that H_2_O_2_ is a key biologically active form of ROS during hypoxia (Liu et al., 2008). ATF4 augments HIF-1α-mediated upregulation of its downstream targets to promote cell survival (Pereira et al., 2014).

Transient exposure to ER stress can condition and prepare cells for survival during a subsequent, more severe stress. This preconditioning is likely due to induction of pro-survival genes, and the ISR is an important prosurvival mechanism under hypoxia. Tumor cells in the primary tumor are exposed to hypoxia and might be preconditioned to survive the subsequent metastatic process. Indeed, cells with compromised PERK-eIF2α-ATF4 signaling are more sensitive to hypoxic stress *in vitro* and they form slower growing tumors *in vivo*, indicating that the PERK-eIF2α-ATF4 pathway confers a survival advantage for tumor cells under hypoxia (Fels and Koumenis, 2006). Severe hypoxia (<0.01%) and ER stress induce in cancer cells the transcription of *ULK1, LC3B*, and *ATG5* through the activity of ATF4 (Rouschop et al., 2010; Pike et al., 2013), and this up-regulation is crucial for maintaining high levels of autophagic flux to survive intratumoral hypoxia, metabolic stress, and starvation.

Analysis of gene expression changes during hypoxia indicated that UPR genes, including genes specifically regulated by XBP1, were most robustly induced during severe hypoxia/anoxia (Romero-Ramirez et al., 2004). Hypoxia induced in a HIF-1α-independent manner *XBP1* expression and activated splicing of its mRNA, resulting in increased levels of XBP1s (Romero-Ramirez et al., 2004). XBP1s colocalizes with hypoxia markers in tumors, and loss of *XBP1* increases the sensitivity of transformed cells to hypoxia-induced apoptosis and inhibits tumor growth (Wouters and Koritzinsky, 2008; Spiotto et al., 2010).

XBP1 is activated in triple-negative breast cancer (TNBC)—a type of breast cancer that does not have estrogen, progesterone and HER2 receptors—and has a pivotal role in the tumorigenicity and progression of TNBC (Chen et al., 2014b). HIF-1α is hyperactivated in TNBCs, but *XBP1* splicing is not directly regulated by HIF-1α. XBP1 drives TNBC tumorigenicity by assembling a transcriptional complex with HIF-1α that augments HIF-1α activity and regulates the HIF-1α transcriptional program, and *XBP1* knockdown reduces mammosphere formation in hypoxic conditions (Chen et al., 2014b). The XBP1 gene expression signature of TNBC patients correlates with HIF-1α and hypoxia-driven signatures and is associated with poor prognosis.

Although it is expected, a connection between ATF6 and hypoxia has not been reported yet and remains largely unexplored. One study showed that ATF6 is activated independently of HIF-1α by simulated ischemia (0.1% O_2_) in a primary cardiac myocyte model system and inactivated upon reperfusion (Doroudgar et al., 2009). The absence of HIF-1α activation at 0.1% O_2_ is consistent with other studies showing that HIF-α activation is maximal at 0.5% O_2_ but decreases to nearly basal levels at lower O_2_ concentrations (Jiang et al., 1996). Furthermore, while PERK and XBP1 activation occur in a HIF-1α-independent manner, a possible involvement of HIF-2α in UPR activation has not been addressed yet.

## Peroxisomes and HIF signaling

### Peroxisomal metabolism and oxygen

**Figure 5:**
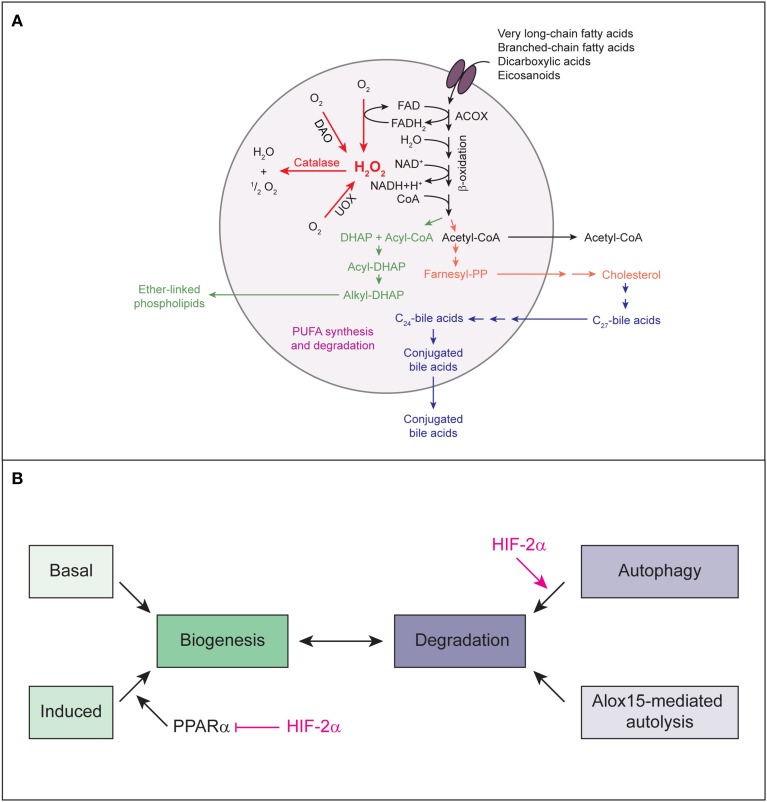
**(A)** The major metabolic pathways in peroxisomes of the mammalian liver. Various lipids are transported by PMPs (e.g., the ABC transporter proteins ABCD1, ABCD2, ABCD3) into the peroxisomal matrix, where they are oxidized by the β-oxidation enzymes. The products of the β-oxidation can serve as substrates for the biosynthesis of ether-linked phospholipids, cholesterol and bile acids or may exit the peroxisome for further oxidation in mitochondria. With regard to PUFAs, peroxisomes not only degrade these compounds but are also involved in their formation through retroconversion of PUFAs by catalyzing the chain-shortening steps. Peroxisomal function depends highly on molecular O_2_ due to its oxidative type of metabolism. Peroxisomal β-oxidation and the activity of other peroxisomal oxidases (e.g., UOX, DAO) result in the production of H_2_O_2_, which is decomposed by catalase. Modified from Schrader and Fahimi (2008). **(B)** Model for HIF-2α-mediated decrease in peroxisome abundance. Peroxisome homeostasis is achieved by balancing biogenesis and degradation of peroxisomes. HIF-2α signaling promotes degradation of peroxisomes by pexophagy. Reduced peroxisome abundance and the ensuing deficiency in peroxisomal function leads to major changes in the lipid profile, such as accumulation of VLCFAs. VLCFAs are activating ligands for the transcription factor PPARα. HIF-2α represses ligand-induced PPARα-mediated peroxisome proliferation and consequential restoration of peroxisome homeostasis. Thus, by simultaneously inducing pexophagy and counteracting PPARα, HIF-2α ensures efficient depletion of the peroxisome pool.

Peroxisomes are ubiquitous and highly dynamic organelles whose number, size, and function are dependent on cell type and metabolic needs. They play key roles in the degradation of fatty acids [i.e., very long-chain fatty acids (VLCFAs), branched-chain FAs, polyunsaturated FAs (PUFAs)], ether lipid synthesis, cholesterol and bile acid synthesis, and metabolism of ROS (Figure 5A) (Van Veldhoven, 2010; Fransen et al., 2012; Faust and Kovacs, 2014). They also act as intracellular signaling platforms in redox, lipid, inflammatory, and innate immunity signaling (Dixit et al., 2010; Nordgren and Fransen, 2014; Odendall et al., 2014). The importance of peroxisomes for cellular metabolism is illustrated by the marked abnormalities in brain and systemic organs in peroxisome biogenesis disorders of the Zellweger spectrum in which functional peroxisomes are absent and disorders caused by single peroxisomal enzyme deficiencies (Raymond et al., 2009). Lack of peroxisomal metabolism creates severe biochemical abnormalities, leading to a variety of clinical symptoms both in patients with peroxisomal disorders as well as peroxisome-deficient mice (Kovacs et al., 2002; Raymond et al., 2009; Baes and Van Veldhoven, 2012; Faust and Kovacs, 2014).

Peroxisomal function depends highly on molecular O_2_ due to its oxidative type of metabolism (Figure 5A). In fact, peroxisomes may be responsible for as much as 20% of O_2_ consumption and 35% of H_2_O_2_ production in tissues such as the liver (Fransen et al., 2012). The number of peroxisomes is approximately 10–15 times less than that of mitochondria (De Duve and Baudhuin, 1966); therefore, on a per unit basis, peroxisomes may consume a significant amount of O_2_ as compared to mitochondria. However, so far there has been no evidence linking HIF signaling to peroxisomes. We hypothesized that to minimize O_2_ consumption under hypoxic conditions, HIF-α signaling may inhibit O_2_-dependent peroxisomal metabolism and/or decrease peroxisome abundance. Since peroxisomes are highly abundant in the liver and liver-specific loss of *Vhl* causes severe lipid accumulation, we investigated peroxisome homeostasis and metabolism in the liver of control and liver-specific *Vhl, Vhl/Hif1*α, and *Vhl/Hif2*α knockout mice and explored the role of HIF-1α and HIF-2α in this context.

### Peroxisome biogenesis and hepatic HIF-α signaling

Peroxisome homeostasis is maintained by balancing biogenesis and degradation of peroxisomes. Peroxisomes can either multiply by growth and fission of pre-existing ones (Schrader et al., 2012) or develop *de novo* from the ER (Tabak et al., 2013). Proteins involved in peroxisome biogenesis, the peroxins, are encoded by *PEX* genes (Hasan et al., 2013; Smith and Aitchison, 2013). In mammalian cells, peroxisome proliferation is triggered by lipids which are substrates of peroxisomal metabolism and ligands of PPARα (Schrader et al., 2012). The majority of peroxins is not induced transcriptionally through peroxisome proliferators. The peroxins PEX11α, β, and γ are involved in the regulation of peroxisome size and number in mammalian cells (Schrader et al., 2012), but only *PEX11*α is a PPARα target gene. Their overexpression increases peroxisome number in the absence of extracellular stimuli or peroxisome metabolism (Li and Gould, 2002; Schrader et al., 2012). Recently, we showed that activation of HIF-α signaling in the liver does not affect the expression of *Pex* genes (Walter et al., 2014). *Pex11*α is the only peroxin that is transcriptionally induced in response to HIF-2α activation in *Vhl*^−∕−^ and *Vhl*^−∕−^*/Hif1*α^−∕−^ livers (Walter et al., 2014). The peroxisome biogenesis machinery in *Vhl*^−∕−^ livers is functional, because peroxisome proliferation can be induced by treatment with PPARα-dependent and -independent peroxisome proliferators. However, HIF-2α signaling has a repressive effect on ligand- and fasting-induced PPARα activation (Figure 5B) (Walter et al., 2014).

### Pexophagy

Three mechanisms for mammalian peroxisome degradation have been described, which include selective autophagy (pexophagy), proteolysis by peroxisomal Lon protease 2 (LONP2), and 15-lipoxygenase-1 (ALOX15)-mediated autolysis (Till et al., 2012). Studies using liver-specific *Atg7*^−∕−^ mice suggest that 70–80% of excess liver peroxisomes are degraded by pexophagy, while the remaining 20–30% are degraded via the action of LONP2 and ALOX15 (Till et al., 2012). A general rule applicable for both yeast and mammalian cells is that environmental conditions that require peroxisomal metabolism lead to peroxisome proliferation, followed by pexophagic degradation when the organelles are no longer required (Iwata et al., 2006; Farré et al., 2008; Motley et al., 2012).

The pexophagy receptors Atg30 and Atg36 were identified in *P. pastoris* and *S. cerevisiae*, respectively, and their overexpression stimulates pexophagy even under peroxisome-inducing conditions (Farré et al., 2008; Motley et al., 2012). Their synthesis is upregulated in peroxisome proliferation conditions, they localize to the peroxisome membrane and bind to Pex3, and they depend on phosphoregulation for their interactions with components of the autophagy machinery (i.e., Atg8, Atg11) during pexophagy conditions (Farré et al., 2008, 2013). Pex3 acts as a docking station for several proteins involved in peroxisomal biogenesis and its interaction with Atg30 regulates the phosphorylation status of Atg30 by a yet unknown kinase (Burnett et al., 2015). Atg30 interacts also with Pex14, the scaffold protein Atg17, and the acyl-CoA binding protein Atg37 (Nazarko et al., 2014). The human ortholog of Atg37, acyl-CoA-binding domain containing protein 5 (ACBD5), is also peroxisomal and required for pexophagy (Nazarko et al., 2014). Despite their functional similarities Atg30 and Atg36 do not display any significant sequence homology and they are conserved only among a few yeast species.

There are no orthologous genes of *Atg30* and *Atg36* in mammals. Overexpression of *NBR1* and *SQSTM1*, which are autophagy receptors of ubiquitinated targets, induces clustering and degradation of peroxisomes in cell lines (Deosaran et al., 2013). SQSTM1 is not required for pexophagy when NBR1 is in excess, but its binding to NBR1 increases the efficiency of NBR1-mediated pexophagy (Deosaran et al., 2013). Artificial mono-ubiquitination of peroxisomal membrane proteins (PMPs) in mammalian cells causes peroxisome turnover by pexophagy in a SQSTM1-dependent manner (Kim et al., 2008). However, it is unknown if a PMP is ubiquitinated under pexophagy-inducing conditions and whether subsequent interaction with NBR1 and/or SQSTM1 links ubiquitinated peroxisomes to the autophagic machinery.

In Chinese hamster ovary (CHO) cells, it has been suggested that under nutrient starvation PEX14 is involved in pexophagy by interacting with the lipidated form of LC3 (Hara-Kuge and Fujiki, 2008). Cell-free synthesized lipidated LC3 interacts in an *in vitro* assay with the transmembrane domain of recombinant PEX14, although PEX14 does not contain a LIR sequence that could ensure LC3 binding (Jiang et al., 2015). PEX14 is an essential component of the peroxisomal translocon complex (Hasan et al., 2013), and it has been proposed that the PEX14-LC3 and PEX14-PEX5 interactions are mutually exclusive (Hara-Kuge and Fujiki, 2008). This competitive interaction might ensure functional segregation of metabolically active and degradation-prone peroxisomes.

Overexpression of *Pex3* in CHO cells and mouse embryonic fibroblasts induced clustering of peroxisomes and NBR1-mediated pexophagy, albeit no direct interaction between PEX3 and NBR1 could be detected (Yamashita et al., 2014). Interestingly, ubiquitin signals were observed on peroxisomes upon *Pex3* overexpression, suggesting that a currently unidentified PMP is ubiquitinated in PEX3-mediated pexophagy and might function in NBR1 recruitment (Yamashita et al., 2014).

### HIF-2α-mediated pexophagy in the liver

We examined the effect of HIF-α signaling on hepatic peroxisome abundance in control and liver-specific *Vhl*^−∕−^, *Vhl*^−∕−^*/Hif1*α^−∕−^, and *Vhl*^−∕−^*/Hif2*α^−∕−^ mice. Peroxisome abundance is significantly decreased in livers of *Vhl*^−∕−^ mice (Walter et al., 2014). Reduction of peroxisome abundance is mediated by HIF-2α, because, with respect to the peroxisomal phenotype, we observe a striking rescue in *Vhl*^−∕−^*/Hif2*α^−∕−^ but not *Vhl*^−∕−^*/Hif1*α^−∕−^ mice. HIF-2α promotes pexophagy because peroxisome abundance is increased after inhibition of autophagy with 3-methyladenine (3-MA) in *Vhl*^−∕−^ mice. In addition, expression of a non-degradable active HIF-2α variant fails to decrease peroxisome abundance in liver-specific, autophagy-deficient *Atg7*^−∕−^ mice (Figure 5B) (Walter et al., 2014). In support of this finding super-resolution and electron microscopy demonstrated that both single and multiple peroxisomes, but no other cytoplasmic organelles, are sequestered in autophagosomes in *Vhl*^−∕−^ livers.

Peroxisome abundance and protein levels of NBR1 and SQSTM1 are concomitantly decreased in *Vhl*^−∕−^ and *Vhl*^−∕−^*/Hif1*α^−∕−^ livers (Walter et al., 2014). Neither peroxisome abundance nor NBR1 and SQSTM1 levels decline in 3-MA-treated *Vhl*^−∕−^ mice, showing that the abundance of these receptors and peroxisomes are interconnected. Expression of a constitutively active HIF-2α variant results also in a concomitant decrease of peroxisome abundance and NBR1 levels. NBR1 and SQSTM1 colocalize with peroxisomes in *Vhl*^−∕−^ livers, but surprisingly NBR1 already localizes to peroxisomes in control livers (Walter et al., 2014). An intriguing feature of autophagy receptors is their tendency to oligomerize, which facilitates sequestration and clustering of the autophagic cargo. Indeed, treatment of *Vhl*^−∕−^ mice with 3-MA leads to a significant clustering of NBR1- and SQSTM1-positive peroxisomes, suggesting that binding of multiple NBR1 and SQSTM1 to peroxisomes and oligomerization of these receptors might induce peroxisome clustering and prime peroxisomes for pexophagy.

In summary, by simultaneously inducing pexophagy and counteracting PPARα, HIF-2α ensures efficient depletion of the peroxisome pool (Figure 5B). Our data show that the autophagy receptors NBR1 and SQSTM1 localize to peroxisomes and are degraded together with peroxisomes by HIF-2α-mediated pexophagy. However, it remains an open question how HIF-2α induces pexophagy at the molecular level, but several possibilities exist and are discussed below.

### Peroxisome abundance in tumors

Peroxisome proliferation is a unique phenomenon generated by a broad spectrum of structurally diverse compounds, such as lipid-lowering drugs and plasticizers (Pyper et al., 2010; Misra et al., 2013). These compounds induce peroxisome proliferation in liver parenchymal cells of rodents, whereas no effects have been observed in non-human primates and humans. Prolonged exposure to peroxisome proliferators leads to the development of hepatocellular carcinomas in rodents (Misra et al., 2013). The mechanism(s) by which these non-mutagenic peroxisome proliferators induce liver tumors remains controversial. Evidence strongly implicates that hyperactivation of PPARα leads to disproportionate large increases in H_2_O_2_-generating enzymes, whereas the expression of H_2_O_2_-degrading enzymes is only moderately increased (Pyper et al., 2010). This imbalance increases the levels of H_2_O_2_ and other ROS in hepatocytes and contributes to oxidative stress, lipid peroxidation, and oxidative DNA damage (Pyper et al., 2010; Misra et al., 2013).

Information on the role of peroxisomes in human tumor development is scarce. It has been shown that the protein levels of peroxisomal branched-chain FA β-oxidation enzymes (i.e., α-methylacyl-CoA racemase, peroxisomal multifunctional protein 2) are upregulated in human prostate cancer (Zha et al., 2005) and that this pathway is essential for optimal proliferation of some prostate cancer cell lines (Zha et al., 2003). Recently, it has been shown that monocarboxylate transporter 2 localizes to peroxisomes in prostate cancer cells and that its expression increases from non-malignant to malignant cells (Valença et al., 2015). It would be important to know if these proteins are selectively upregulated or if peroxisome abundance is also increased in prostate cancer.

A decrease in peroxisome abundance has been observed in various tumor cells, including hepatocellular carcinoma (Litwin et al., 1999), colon carcinoma (Cable et al., 1992; Lauer et al., 1999), breast cancer (el Bouhtoury et al., 1992; Keller et al., 1993), and in renal cell carcinoma (Frederiks et al., 2010). However, so far the mechanism leading to reduced peroxisome abundance was unknown. We explored the relevance of HIF-2α-dependent pexophagy in human clear cell renal cell carcinomas (ccRCC), because loss of *VHL* function occurs in up to 90% of sporadic human ccRCC and HIF-2α is considered to be a driver oncoprotein for ccRCC. Analysis of more than 200 ccRCC tissue samples revealed that peroxisome abundance is reduced in *VHL*-deficient ccRCC characterized by high HIF-2α levels (Walter et al., 2014), suggesting that HIF-2α-mediated pexophagy is relevant to human disease. Interestingly, peroxisome abundance is reduced more frequently in well-differentiated tumors, however, it remains to be determined if induction of pexophagy and subsequent loss of peroxisomes promotes or slows down tumor growth. Since HIF-2α stabilization is observed in the vast majority of solid tumors (Franovic et al., 2009; Qing and Simon, 2009), we propose that in addition to ccRCC HIF-2α-mediated pexophagy might also lead to reduced peroxisome abundance in other cancer types.

### Metabolic consequences of reduced peroxisome abundance

Livers of *Vhl*^−∕−^ and *Vhl*^−∕−^*/Hif1*α^−∕−^ mice are enlarged and display severe steatosis (Rankin et al., 2009; Walter et al., 2014). HIF-2α-mediated reduced peroxisome abundance leads to major changes in the lipid profile of *Vhl*^−∕−^ and *Vhl*^−∕−^*/Hif1*α^−∕−^ livers, like accumulation of VLCFAs and VLC-PUFAs and depletion of docosahexaenoic acid (DHA) and arachidonic acid (Walter et al., 2014). Furthermore, the levels of the C_27_-bile acid intermediates 3α,7α-dihydroxycholestanoic acid and 3α,7α,12α-trihydroxycholestanoic acid are significantly increased in the plasma of *Vhl*^−∕−^ and *Vhl*^−∕−^*/Hif1*α^−∕−^ mice. These lipid changes are characteristic features of human patients and mice lacking peroxisomes (Raymond et al., 2009; Van Veldhoven, 2010; Wanders et al., 2010; Baes and Van Veldhoven, 2012). β-oxidation of VLCFAs and C_27_-bile acid intermediates occurs only in peroxisomes, peroxisomes play a role in synthesis and degradation of PUFAs, and DHA synthesis requires one cyle of peroxisomal β-oxidation (Van Veldhoven, 2010). Since peroxisomes are essential for plasmalogen biosynthesis and substrates for peroxisomal β-oxidation also include branched-chain FAs, dicarboxylic FAs, and eicosanoids (Van Veldhoven, 2010), it is likely that additional changes in lipid metabolism result from reduced peroxisome abundance in response to HIF-2α activation.

Indirect consequences of reduced peroxisomal metabolism like activation of ER stress pathways and mitochondrial dysfunction might also contribute to alterations in lipid metabolism (Baumgart et al., 2001; Dirkx et al., 2005; Kovacs et al., 2009, 2012). Toxic effects of accumulation of peroxisomal β-oxidation substrates might damage the mitochondrial compartment by altering the lipid composition of mitochondrial membranes. It is well-known that free fatty acids act as potent detergents that can damage cellular membranes (Ho et al., 1995). Membrane properties (e.g., acyl chain order, fluidity, permeability, fusion events, lipid raft microdomains, protein activity) are affected by changes in VLCFAs, VLC-PUFAs, DHA, and plasmalogen levels, influencing secretory and vesicular trafficking pathways (Whitcomb et al., 1988; David et al., 1998; Gleissman et al., 2010; Obara et al., 2013). Hypoxia or loss of VHL function has been shown to delay endocytosis and thereby to enhance receptor tyrosine kinase-mediated signaling (Wang et al., 2009). Thus, lipid alterations as a result of HIF-2α-mediated pexophagy might affect endosomal trafficking and signaling pathways downstream of membrane receptors.

A hallmark of cancer is the reprogramming of metabolism, and recent data suggest that alterations in lipid metabolism play an important role in tumor development (Hirsch et al., 2010; Santos and Schulze, 2012; Currie et al., 2013). Fatty acids support cancer growth by providing substrates for energy production or by generating building blocks for membranes and signaling lipids in proliferating cells (Carracedo et al., 2013). VLC-PUFAs could be converted to eicosanoids, biologically active lipids involved in various pathological processes such as inflammation and cancer. Eicosanoids are degraded in peroxisomes, and loss of peroxisomes affects eicosanoid signaling. Peroxisomes are essential for the synthesis of ether lipids, which represent up to 20% of the total phospholipid mass in humans (Braverman and Moser, 2012; Lodhi and Semenkovich, 2014). Aggressive cancers have high levels of ether lipids, and the expression of the peroxisomal ether lipid synthetic enzyme alkylglyceronephosphate synthase (AGPS) is increased in various cancer cell lines and primary tumors (Benjamin et al., 2013). AGPS knockdown impairs cancer pathogenesis through not only lowering the levels of ether lipids, but also by altering fatty acid, eicosanoid, and glycerophospholipid metabolism, resulting in an overall reduction in the levels of several oncogenic signaling lipids (Benjamin et al., 2013).

Several environmental challenges including ischemia-reperfusion injury, obstructive sleep apnea, viral hepatitis, and alcohol-mediated liver injury are known to induce hepatic hypoxia signaling and are associated with changes in lipid metabolism (Nath and Szabo, 2012). It is tempting to speculate that HIF-2α-mediated pexophagy contributes, at least in some of these pathophysiological conditions, to alterations in lipid metabolism. Decreased plasma and hepatic levels of arachidonic acid and DHA have been observed in patients with non-alcoholic fatty liver disease and non-alcoholic steatohepatitis (Puri et al., 2007, 2009), suggesting impaired peroxisomal metabolism in their pathogenesis. An increasing number of studies suggest that peroxisome dysfunction may be a specific marker for Alzheimer disease. Kou et al. (2011) noted extensive peroxisome-related alterations in Alzheimer disease brains such as increased VLCFAs and decreased plasmalogens containing PUFAs. The question remains if the general loss of peroxisome functions in AD brains is due to pexophagy.

### Models how HIF-2α might trigger pexophagy

**Figure 6:**
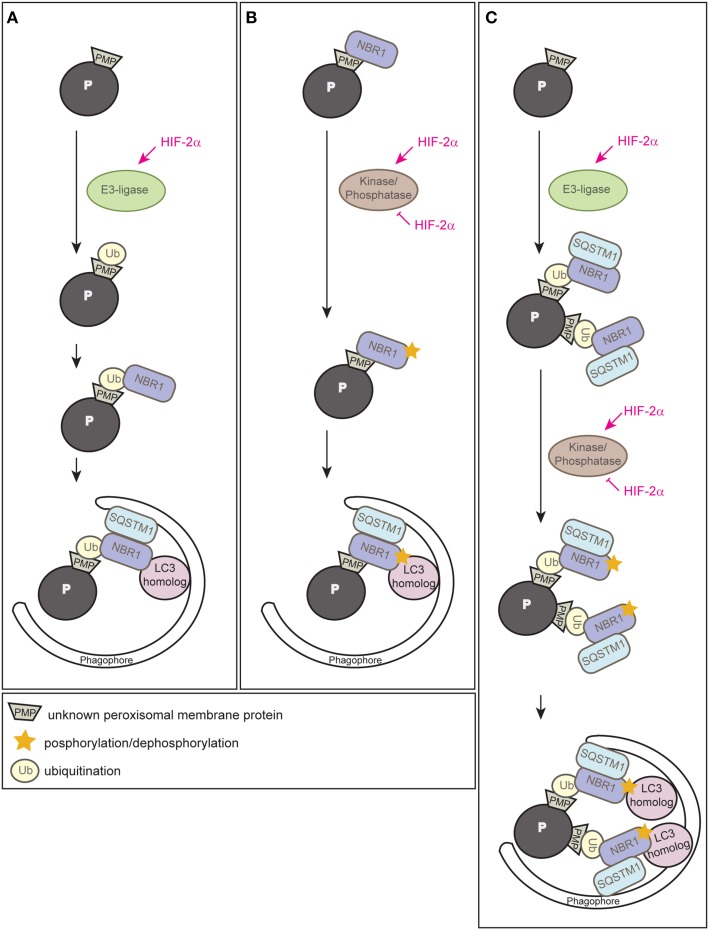
**Three alternative models illustrating how HIF-2α might trigger pexophagy. (A)** HIF-2α might induce an E3 ubiquitin ligase that ubiquitinates a PMP that enhances the recruitment of the autophagy receptor NBR1 to the peroxisome surface. Accumulation of NBR1 on peroxisomes likely recruits SQSTM1, which was suggested to act as pexophagy co-receptor, and subsequently leads to clustering of peroxisomes via oligomerization of receptor-bound organelles. Accumulation of a critical mass of autophagy receptors might prime phagophore assembly at peroxisomes. **(B)** NBR1 could be recruited to peroxisomes independently of ubiquitin via its membrane-interacting amphipathic α-helical J domain. HIF-2α might induce or inhibit a kinase/phosphatase that leads to a change in the posttranslational modification of peroxisome-bound NBR1 and thereby triggers recruitment of the autophagic machinery. **(C)** HIF-2α-dependent activation of pexophagy might be a 2-step process and HIF-2α functions as master regulator that combines two layers of posttranslational modifications to trigger pexophagy. First, it induces an E3 ubiquitin ligase leading to an increased ubiquitination of PMP(s) and subsequent accumulation of NBR1 and SQSTM1 on peroxisomes. Second, HIF-2α activates or inhibits a kinase/phosphatase that leads to a change in the posttranslational modification of peroxisome-bound NBR1 and thereby enhances its binding affinity to a LC3 homolog that finally results in pexophagy. Modified from Schönenberger et al. (2015).

Since HIF-2α is a transcription factor, the most likely possibility would be that HIF-2α induces the expression of an autophagy receptor and subsequent clustering of peroxisomes via oligomerization of receptor-bound organelles, however, neither *Nbr1* nor *Sqstm1* are HIF-2α target genes (Walter et al., 2014). Ubiquitination of cargo prone for selective autophagic degradation is the most prevalent autophagy-targeting signal in mammals, and most of the currently known autophagy receptors harbor both ubiquitin-binding domains and LIRs (Kirkin et al., 2009; Stolz et al., 2014). HIF-2α might induce an E3 ubiquitin ligase that mediates the ubiquitination of a PMP (Figure 6A). We propose that HIF-2α signaling increases in this manner NBR1 accumulation on peroxisomes, which in turn serves as a platform for the recruitment of SQSTM1 to achieve a critical mass of autophagy receptors on peroxisomes required for pexophagy (Schönenberger et al., 2015). This might concentrate sufficient ubiquitin-like modifiers (e.g., LC3 and GABARAPs) in close proximity to peroxisomes to prime phagophore assembly. The peroxisomal membrane harbors three E3 ligases (PEX2, PEX10, PEX12) that are essential for peroxisome biogenesis and involved in PEX5 receptor ubiquitination. Their transcriptional induction and concomitant increase of protein levels could increase their ubiquitination capability leading to enhanced ubiquitination of PMPs, but HIF-2α does not induce the expression of those E3 ligases (Walter et al., 2014). Thus, further studies are required to identify and characterize putative E3 ligases involved in HIF-2α-mediated pexophagy.

Why does NBR1 localize to peroxisomes in control livers where pexophagy is not induced? In fact, yeast Atg30 and Atg36 also localize to peroxisomes under peroxisome proliferation conditions, but they depend on phosphoregulation for their interactions with components of the autophagy machinery during pexophagy conditions (Farré et al., 2008, 2013). Posttranslational modifications (e.g., phosphorylation, ubiquitination, acetylation) of autophagy proteins are crucial for induction, inhibition, cargo-recognition, and fine-tuning of autophagy. We propose that additional protein modifications are very likely necessary to ultimately drive pexophagy by recruiting and tailoring the autophagic machinery to peroxisomes. For example, phosphorylation as an inducing event of autophagy is conserved from yeast to mammals and has already been discussed above in the context of pexophagy in yeast and mitophagy. Phosphorylation of SQSTM1 and optineurin increases affinity to ubiquitin chains and LC3 (Stolz et al., 2014), and NBR1 phosphorylation by glycogen synthase kinase 3 prevents the aggregation of ubiquitinated proteins and their selective autophagic degradation (Nicot et al., 2014). We propose that HIF-2α governs pexophagy by promoting posttranslational modifications of PMPs and/or autophagy receptors that enhance interactions of receptor-labeled peroxisomes with the autophagic machinery (Figure 6B) (Schönenberger et al., 2015).

Finally, one could envision that HIF-2α-dependent activation of pexophagy is a 2-step process that involves interplay between ubiquitination of a PMP(s) as well as phosphoregulation of autophagy receptors or a PMP(s) as a trigger of pexophagy (Figure 6C) (Schönenberger et al., 2015). Interestingly, a similar interplay promotes PINK1-Parkin-mediated mitophagy whereby phosphorylation of ubiquitin contributes to a feedforward mechanism for ubiquitination events on dysfunctional mitochondria (Ordureau et al., 2014).

A receptor protein complex (RPC) model has been proposed that encompasses the receptor protein as the key player that establishes interactions with ligands, scaffold, and phagophore proteins (Nazarko et al., 2014). The question remains which components of the RPC are involved in HIF-2α-driven pexophagy. Is there an Atg11 homolog in the mammalian liver that would act as a scaffold for HIF-2α-mediated pexophagy? Little is known about mammalian autophagy adaptor proteins that bind to LC3 family members and serve as an anchor point to regulate autophagosome formation around the specific cargo (Stolz et al., 2014). Similar to Atg11, ALFY (autophagy-linked FYVE protein) is a scaffolding protein implicated in aggrephagy that links cargo to the autophagic machinery (Isakson et al., 2013). Moreover, Huntingtin (HTT) has been proposed to serve as adaptor for any type of selective autophagy, because the domain of HTT shares structure similarities and binding activity with the yeast Atg11 protein and interacts with autophagic effector proteins (Ochaba et al., 2014). It is tempting to speculate that ALFY or HTT are part of the RPC mediating HIF-2α-induced pexophagy and thus, functioning as scaffold protein(s). In summary, the identification of HIF-2α as an inducer of pexophagy opens new avenues for studying the underlying molecular mechanism.

## Concluding remarks

We have described hypoxia signaling pathways that regulate function and abundance of mitochondria, ER, and peroxisomes under hypoxia or in response to loss of VHL function. There is emerging evidence that these O_2_-related organelles exhibit a close functional interplay, and peroxisomal alterations influence mitochondrial and ER functions and vice versa. Although peroxisomal function depends highly on molecular O_2_, there has been no evidence linking their abundance to O_2_ availability and HIF-α signaling. In a recent study we identified a unique function of HIF-2α as promoter of pexophagy. An open question that remains to be answered is how HIF-2α induces pexophagy, and we discussed in this review alternative models for how it might trigger pexophagy. Posttranslational modification of autophagy-related proteins and receptors has emerged as an essential regulatory mechanism of selective autophagy. Future studies should address which posttranslational modifications regulate HIF-2α-mediated pexophagy and which components of the receptor protein complex are involved in HIF-2α-mediated pexophagy. In addition, it remains to be determined how HIF-α signaling affects mitochondrial size and ultrastructure and consequently their activity. PPARα modulates metabolic and inflammatory pathways by responding to nutritional signals through ligand activation of transcription, and it is a target of drugs in use and in development to treat diseases. We showed that HIF-α signaling has a repressive effect on ligand-dependent PPARα transcriptional activity, but the mechanism by which HIF-α exerts its inhibitory effect requires further studies. In the past the role of peroxisomes in the cell and in human disease apart from peroxisomal disorders has been grossly underestimated, but this might change given increasing appreciation for the complexity of their interactions with other organelles and the recent discovery of novel functions for peroxisomes. Reduction in peroxisome abundance by pexophagy might positively and negatively impact human disorders including cancer, inflammation, metabolic and neurodegenerative diseases. Along with mechanistic studies of HIF-2α-dependent regulation of pexophagy, the identification of pharmacological regulators of pexophagy might have practical health benefits.

### Conflict of interest statement

The authors declare that the research was conducted in the absence of any commercial or financial relationships that could be construed as a potential conflict of interest.

## Abbreviations

ALFY: autophagy-linked FYVE protein
ALOX15: 15-lipoxygenase-1
ATF: activating transcription factor
Atg: autophagy-related protein
BNIP3: Bcl-2 and adenovirus E1B 19-kDa-interacting protein 3
BNIP3L/NIX: BNIP3-like
ccRCC: clear cell renal cell carcinoma
CHO: Chinese hamster ovary
CHOP: C/EBP homologous protein
CK2: casein kinase 2
COX: cytochrome c oxidase
DAO: D-amino acid oxidase
DHA: docosahexaenoic acid
ER: endoplasmic reticulum
ERAD: ER-associated degradation
ERR: estrogen-related receptor
FA: fatty acid
FOXO3a: forkhead-box protein O3a
FUNDC1: FUN14 domain containing 1
GABARAP: γ-aminobutyric acid-receptor-associated protein
GADD34: growth arrest DNA-inducible gene 34
GLUT: glucose transporter
GRP: glucose-regulated protein
HIF: hypoxia-inducible factor
HTT: Huntingtin
IRE1: inositol-requiring protein 1
LC3: microtubule-associated protein-1 light chain 3
LDHA: lactate dehydrogenase A
LIR: LC3-interacting region
LONP: Lon protease
MAX: MYC-associated factor X
MCT4: monocarboxylate transporter 4
mtROS: mitochondrial reactive oxygen species
MXI1: MAX-interacting protein 1
NBR1: neighbor of BRCA1 gene
NRF: nuclear respiratory factor
OMM: outer mitochondrial membrane
OxPhos/ETC: oxidative phosphorylation and electron transport chain
PDH: pyruvate dehydrogenase
PDK1: pyruvate dehydrogenase kinase 1
PERK: protein kinase RNA-like ER kinase
PEX: peroxin
PGAM5: phosphoglycerate mutase family member 5
PHD: prolyl hydroxylase
PGC-1: PPARγ
coactivator 1
PINK1: PTEN-induced putative protein kinase 1
PMP: peroxisomal membrane protein
PPAR: peroxisome proliferator-activated receptor
PTEN: phosphatase and tensin homolog
PUFA: polyunsaturated fatty acid
ROS: reactive oxygen species
RPC: receptor protein complex
SQSTM1/p62: sequestosome 1
TNBC: triple-negative breast cancer
ULK1: UNC51-like kinase 1
UOX: urate oxidase
UPR: unfolded protein response
VDAC: voltage-dependent anion channel
VEGF: vascular endothelial growth factor
VHL: von Hippel-Lindau
VLC: very long-chain
XBP1: X-box-binding protein 1.
